# Data on Laurdan spectroscopic analyses to compare membrane fluidity between susceptible and multidrug-resistant bacteria

**DOI:** 10.1016/j.dib.2018.09.106

**Published:** 2018-10-02

**Authors:** Lucinda J. Bessa, Mariana Ferreira, Paula Gameiro

**Affiliations:** LAQV/REQUIMTE, Departamento de Química e Bioquímica, Faculdade de Ciências da Universidade do Porto, Porto, Portugal

## Abstract

The data presented are related to the research article entitled “Evaluation of membrane fluidity of multidrug-resistant isolates of *Escherichia coli* and *Staphylococcus aureus* in presence and absence of antibiotics” (Bessa et al., 2018) [1]. This data article provides a dataset that includes emission spectra of Laurdan-labeled bacteria, namely of three *Escherichia coli* and three *Staphylococcus aureus* strains. Laurdan (6-dodecanoyl-2-dimethylaminonaphthalene) is a popular fluorescence probe used to assess membrane fluidity. Laurdan detects changes in membrane phase properties through its sensitivity to the polarity of the environment in the bilayer. Polarity changes are reflected by shifts in the Laurdan fluorescence emission spectrum that can be quantified by calculating the excitation generalized polarization (*GP_exc_*).

## Specifications table

**Table:** 

Subject area	*Biophysics*
More specific subject area	*Membrane fluidity*
Type of data	*Figures, Table and Scheme*
How data was acquired	*Fluorescence spectroscopy using a Varian Cary Eclipse fluorescence spectrofluorometer*
Data format	*Partially analyzed*
Experimental factors	*Exponentially growing cells were used to prepare standard inocula, which were incubated at 37 °C for 6 days. At 24, 48, 72 and 144 h, samples were taken and treated with 0.5 µM Laurdan for 1.5 h at 37 °C with shaking (500 rpm).*
Experimental features	*Laurdan-labeled bacterial samples were transferred to a 1-cm quartz cuvette and Laurdan emission spectra were obtained through the spectrofluorometer.*
Data source location	*Porto, Portugal*
Data accessibility	*Data are available within this article*

## Value of the data

• The data reported here furnishes Laurdan emission spectra of *E. coli* ATCC 25922 and *S. aureus* ATCC 25923 as well as of two multidrug-resistant clinical isolates of each species.
• Allows calculation of excitation *GP* (*GP_exc_*) from the respective spectra using the equation: ${\mathrm{GP}}_{\mathrm{exc}}=\frac{I_{440}-I_{490}}{I_{440}+I_{490}}$, where *I*_440_ and *I*_490_ are fluorescence intensities at 440 and 490 nm, respectively.
• The higher the *GP_exc_* values, the closer the cytoplasmic membranes are to a saturated liquid ordered (Lo) – less fluid, while lower *GP_exc_* values mean membranes in a unsaturated liquid disordered (Ld) phase – more fluid [1], [2], [3].

## 1 Data

**Fig. 1:**
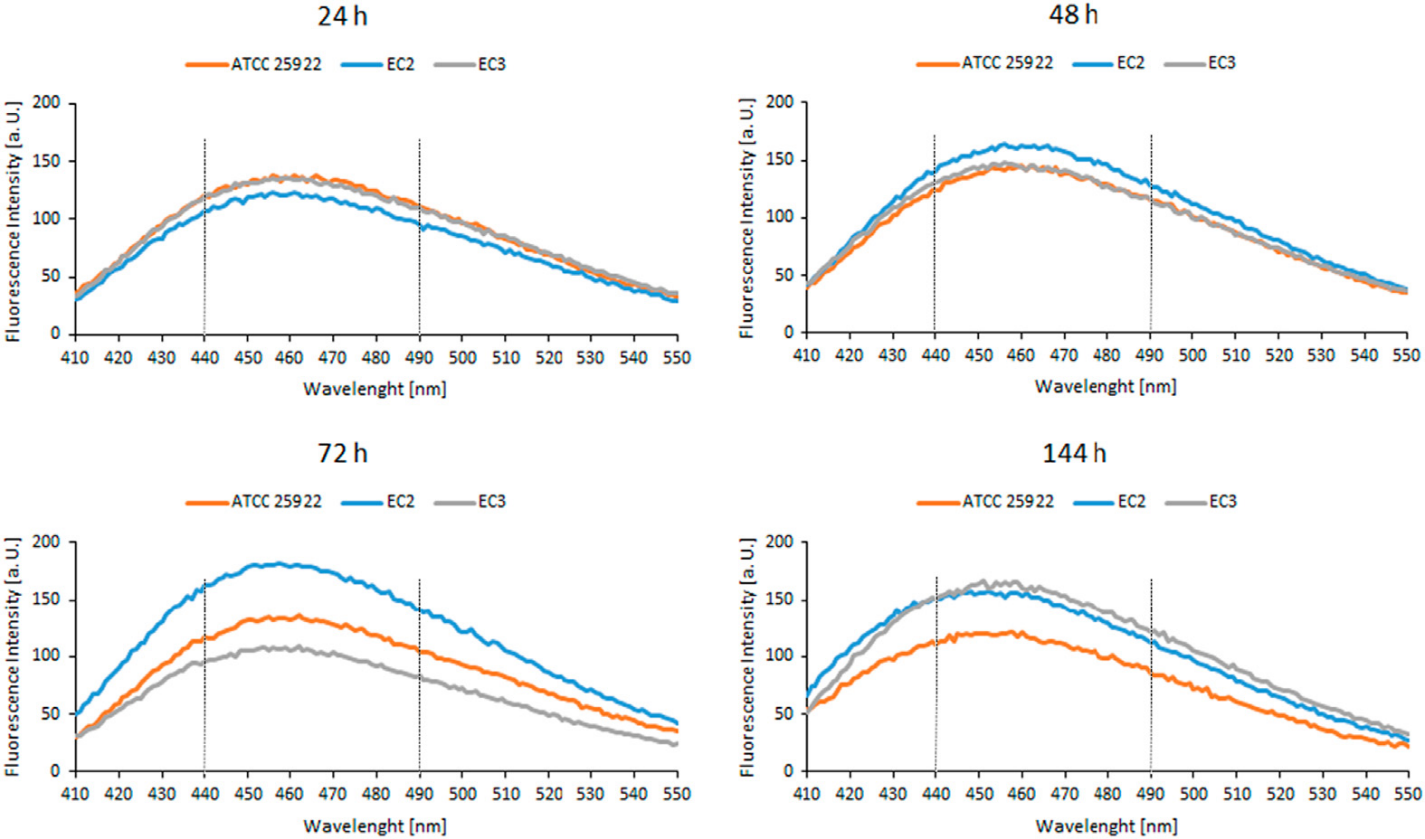
Laurdan emission spectra in three *E. coli* strains – *E. coli* ATCC 25922 and two multidrug-resistant isolates, *E. coli* EC2 and *E. coli* EC3 – at 24, 48, 72 and 144 h after bacterial growth at 37 °C. The excitation wavelength was 350 nm. Data presented are from a single experiment. Intensities at 440 and 490 nm were used to calculate excitation generalized polarization values (*GP_exc_*).

**Fig. 2:**
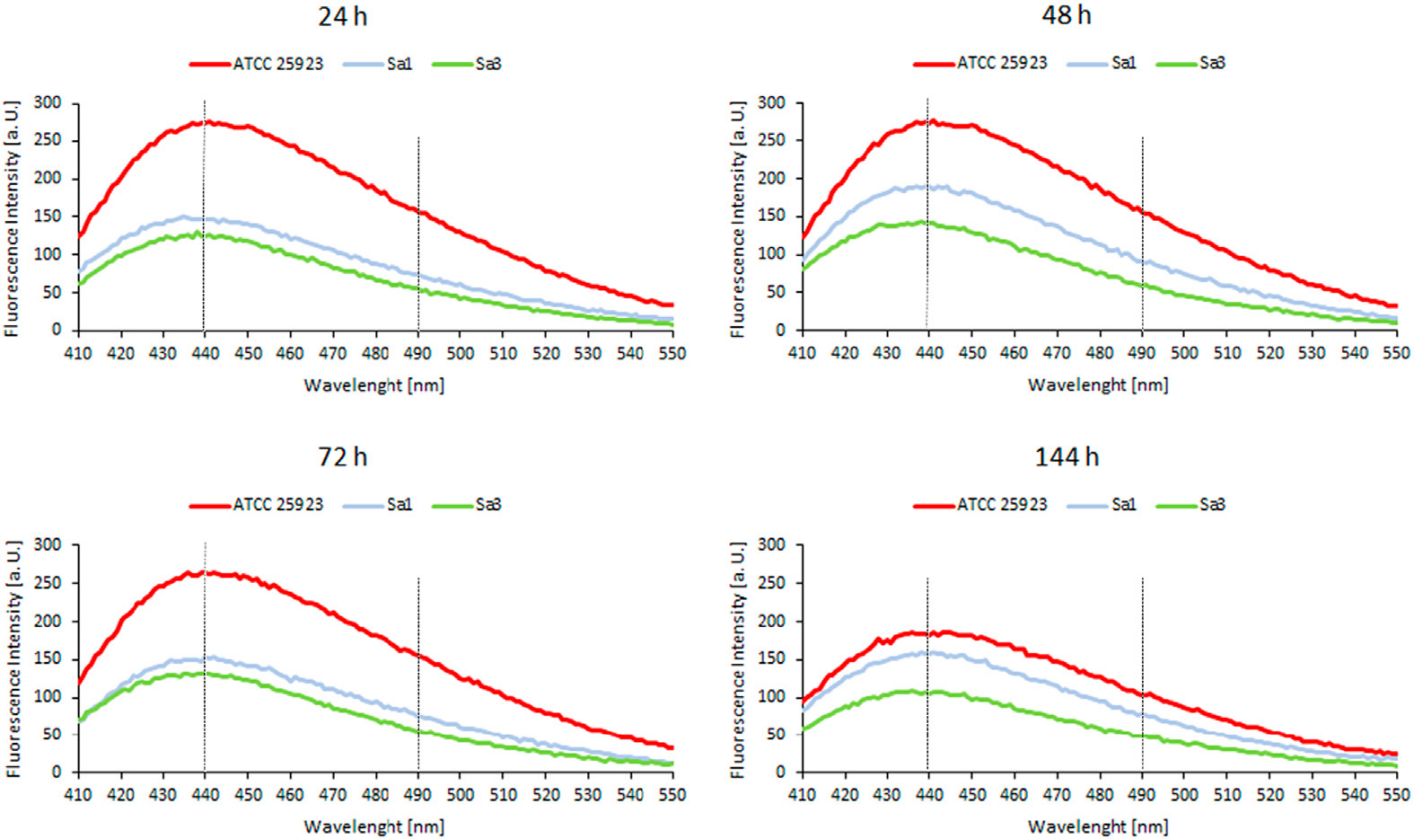
Laurdan emission spectra in three *S. aureus* strains – *S. aureus* ATCC 25923 and two MRSA isolates, *S. aureus* Sa1 and *S. aureus* Sa3 – at 24, 48, 72 and 144 h after bacterial growth at 37 °C. The excitation wavelength was 350 nm. Data presented are from a single experiment. Intensities at 440 and 490 nm were used to calculate excitation generalized polarization values (*GP_exc_*).

The dataset of this article includes the fluorescence emission spectra of Laurdan-labeled bacteria obtained after the subtraction of the respective blank spectra (spectra obtained from unlabeled samples). Spectra were acquired at four time points (24, 48, 72 and 144 h). Data refers to three *E. coli* strains – *E. coli* ATCC 25922 and two multidrug-resistant clinical isolates, *E. coli* EC2 and *E. coli* EC3 (Fig. 1) – and to three *S. aureus* strains – *S. aureus* ATCC 25923 and two methicillin-resistant *S. aureus* (MRSA) isolates, *S. aureus* Sa1 and *S. aureus* Sa3 (Fig. 2). These bacterial strains are described in Ref. [1].

**Scheme 1:**
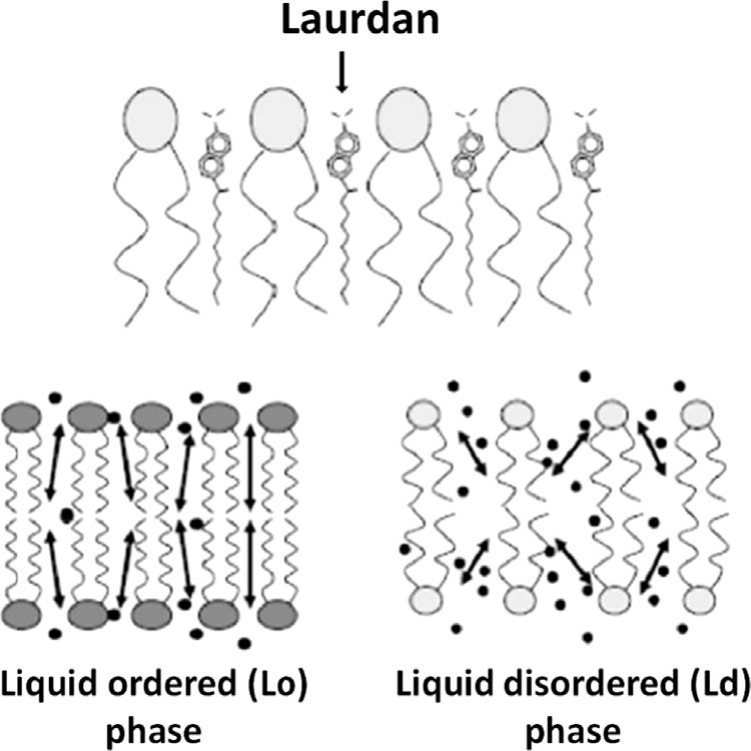
Schematic representation of Laurdan and of its localization (black points) within the phospholipid bilayer in the Lo and Ld phases.

From each spectrum, *GP_exc_* values were obtained (Table 1). *GP*_*exc*_ helps analyzing the spectra by allowing quantification, which facilitates the straight comparison between differing experimental conditions. The standard equation used to calculate *GP_exc_* is: ${\mathrm{GP}}_{\mathrm{exc}}=\frac{I_{440}-I_{490}}{I_{440}+I_{490}}$, where *I*_440_ and *I*_490_ are the fluorescence intensities at 440 and 490 nm, respectively. In the liquid ordered (Lo) phase, Laurdan emission spectrum has a maximum at 490 nm (traditionally called red shift), whereas in the liquid disordered phase (Ld), it has a maximum at 440 nm illustrating a blue shift [2], [4]. *GP_exc_* values found experimentally can range from 0.5 to -0.3; typically *GP_exc_* values in the Ld phase range from 0.3 to -0.3 while in the Lo phase from 0.5 to 0.6 [5]. Scheme 1 illustrates the localization of Laurdan within the phospholipid bilayer either in the Lo phase or in the Ld phase.

**Table 1:** Intensities values at 440 and 490 nm abstracted from Laurdan emission spectra obtained from *E. coli* and *S. aureus* strains throughout time (up to 144 h) and the respective *GP_exc_* values are presented.

	**24 h**			**48 h**			**72 h**			**144 h**		
***E. coli*****strains**	**ATCC 25922**	**EC2**	**EC3**	**ATCC 25922**	**EC2**	**EC3**	**ATCC 25922**	**EC2**	**EC3**	**ATCC 25922**	**EC2**	**EC3**

***I***_**440**_	121.1	106.7	118.8	124.3	139.7	130.3	117.1	163.2	115.2	110.1	150.7	150.3
***I***_**490**_	111.7	94.8	110.4	116.1	127.6	117.3	104.4	141.7	100.4	84.9	112.3	121.7
***GP**_**exc**_*	0.040	0.059	0.037	0.034	0.045	0.052	0.058	0.070	0.068	0.129	0.146	0.105
	**24 h**			**48 h**			**72 h**			**144 h**		
***S. aureus*****strains**	**ATCC 25923**	**Sa1**	**Sa3**	**ATCC 25923**	**Sa1**	**Sa3**	**ATCC 25923**	**Sa1**	**Sa3**	**ATCC 25923**	**Sa1**	**Sa3**

***I***_**440**_	418.5	147.1	123.9	272.9	190.4	141.8	265.3	153.2	131.5	182.9	157.2	104.5
***I***_**490**_	233.7	75.1	55.5	156.3	91.9	59.3	154.8	75.0	55.1	104.2	79.0	48.0
***GP**_**exc**_*	0.283	0.324	0.382	0.272	0.349	0.410	0.263	0.342	0.409	0.274	0.331	0.370

## 2 Experimental design, materials and methods

### 2.1 Strains and culture conditions

*E. coli* ATCC 25922 and *S. aureus* ATCC 25923 were used as reference (susceptible) strains. Two multidrug-resistant clinical isolates of *E. coli*, EC2 and EC3, and two methicillin-resistant *S. aureus* (MRSA) clinical isolates, Sa1 and Sa3, were also used.

Fresh exponentially growing bacterial cells were used to prepare standard inocula (cell suspension with an optical density at 600 nm – *OD*_600_ – of 0.1) in Nutrient Broth (NB – Liofilchem s.r.l., Roseto degli Abruzzi, Italy). All inocula were incubated at 37 °C with shaking (100 rpm) in a B.Braun Certomat WR shaking water bath (BBI Biotech, Berlin, Germany) for up to six days.

### 2.2 Sample preparation for membrane labeling

At 24, 48, 72 and 144 h, samples were taken and subsequently diluted in NB to obtain an *OD*_600_ of 0.4 [6], [7]. From such bacterial suspensions, aliquots of 1.5 mL were centrifuged at 10 000 rpm for 8 min, washed twice in 15 mM Tris–HCl buffer (pH 7.4), and resuspended in 0.5 µM of Laurdan (Sigma-Aldrich, St. Louis, Missouri, USA) from a 0.2 mM stock solution in dimethylformamide. Membrane labeling by Laurdan happened during incubation in the dark, at 37°C with shaking (500 rpm) for 1.5 h.

### 2.3 Laurdan fluorescence measurements

A 1-mL aliquot of unlabeled and labeled samples was transferred to a 1-cm quartz cuvette for further acquisition of Laurdan emission spectra. A Varian Cary Eclipse fluorescence spectrofluorometer (Agilent Technologies, Santa Clara, California, USA) equipped with a temperature controller and a magnetic cuvette stirrer was used. The temperature was set at 37.0 ± 0.1°C.

Emission spectra of Laurdan-labeled bacteria were obtained and recorded through the Cary Eclipse Software at an excitation wavelength of 350 nm using emission wavelengths from 410 to 550 nm. Slit widths of 10 nm were used. Blank samples were also used and their emission spectra were subtracted to the respective sample spectrum. *GP_exc_* was calculated from the equation: ${\mathrm{GP}}_{\mathrm{exc}}=\frac{I_{440}-I_{490}}{I_{440}+I_{490}}$, where *I*_440_ and *I*_490_ indicate fluorescence intensities at 440 and 490 nm, respectively [2].
