# Corrigendum to “Data on Laurdan spectroscopic analyses to compare membrane fluidity between susceptible and multidrug-resistant bacteria” [Data in Brief 21 (2018) 128–132]

**DOI:** 10.1016/j.dib.2019.104296

**Published:** 2019-07-18

**Authors:** Lucinda J. Bessa, Mariana Ferreira, Paula Gameiro

**Affiliations:** Departamento de Química e Bioquímica, Faculdade de Ciências da Universidade do Porto, Porto, Portugal

The authors regret to report a mistake that went unnoticed and ask for a corrigendum to the above mentioned paper. In the sentence (on page 129, section 1.Data): “In the liquid ordered (Lo) phase, Laurdan emission spectrum has a maximum at **490 nm** (traditionally called **red** shift), whereas in the liquid disordered phase (Ld), it has a maximum at **440 nm** illustrating a **blue** shift [2,4].”, the words we highlight now in bold are mismatched. The correct sentence must be < In the liquid ordered (Lo) phase, Laurdan emission spectrum has a maximum at 440 nm (traditionally called blue shift), whereas in the liquid disordered phase (Ld), it has a maximum at 490 nm illustrating a red shift [2,4].>

The authors would like to apologise for any inconvenience caused.

