# Supplementary material for: Data on Laurdan spectroscopic analyses to compare membrane fluidity between susceptible and multidrug-resistant bacteria
Source: Data Brief. 2018 Oct 2;21:128–32. doi: 10.1016/j.dib.2018.09.106 (PMC6187016; doi:10.1016/j.dib.2018.09.106)
Supplement: Supplementary file 1 — Supplementary material [file mmc1.docx]

"Conflict of interest

The authors have no conflict of interest to declare. "
